# Weight Management Programme for Overweight and Obese Adults in Ningbo, China: A Feasibility Pre- and Post-intervention Study

**DOI:** 10.3389/fpubh.2019.00388

**Published:** 2019-12-18

**Authors:** Miao Xu, Kaushik Chattopadhyay, Jialin Li, Nanin Rai, Yanshu Chen, Fangfang Hu, Jianping Chu, Li Li

**Affiliations:** ^1^Department of Endocrinology and Metabolism, Ningbo First Hospital, Ningbo, China; ^2^Division of Epidemiology and Public Health, School of Medicine, University of Nottingham, Nottingham, United Kingdom; ^3^School of Medicine, University of Nottingham, Nottingham, United Kingdom

**Keywords:** China, feasibility study, obesity, overweight, weight management

## Abstract

**Objective:** To investigate the feasibility of conducting a randomized controlled trial (RCT) of a weight management programme in Ningbo, China.

**Methods:** A pre- and post-intervention study was conducted in Ningbo. The study duration was from 01 July 2015 to 30 September 2017. Those aged 18–65 years of age, with a body mass index (BMI) of ≥24 kg/m^2^ (i.e., overweight/obese) and were willing to comply with the weight management programme were included in the study. The programme, focusing on lifestyle modification, was delivered by a multidisciplinary team over a period of 3-months. The study parameters were recruitment rate, follow-up rates at 3- and 6-month and standard deviation (SD) of the outcome measure (i.e., BMI).

**Results:** Out of 261 people who gave written informed consent and were screened for eligibility, 193 (74%) were found eligible and were enrolled on to the programme. Those who enrolled on to the programme, 185 (96%) and 169 (88%) were followed up at 3- and 6-month, respectively. The SD of the outcome measure was 4.85. BMI reduced at 3-month follow-up (−1.98, 95% CI −2.19 to −1.79, *p* < 0.001) and at 6-month follow-up (−2.42, −2.71 to −2.11, <0.001).

**Conclusion:** Based on the promising recruitment and follow-up rates in this study, it would be feasible to undertake the RCT of the weight management programme for overweight and obese adults in Ningbo. The SD of the outcome measure is used to calculate the sample size of the RCT.

**Clinical Trial Registration:** www.ClinicalTrials.gov, identifier: ChiCTR1900025861.

## Introduction

Body mass index (BMI) is one of the most widely used methods to define overweight and obesity in adults (1). Overweight is defined as a BMI of 24.0–27.9 kg/m^2^, and obesity is defined as a BMI of ≥28.0 kg/m^2^ (2). Overweight and obesity is a grave public health challenge, which is rapidly growing in China (3). The prevalence of overweight and obesity among adults in China is around 30 and 12%, respectively (4).

Overweight and obesity can have major health, social, and economic consequences (5, 6). It can lead to a number of serious and potentially life-threatening diseases, such as metabolic syndrome, type-2 diabetes, coronary heart disease, stroke, and some types of cancer (7). The main risk factors associated with it are unhealthy diet and physical inactivity (8). Thus, in overweight and obese adults, weight management interventions are recommended, which focus on lifestyle modification, such as a healthy diet and physical activity (9).

Ningbo is one of the most economically developed Chinese cities, located in the northeast Zhejiang province (10). In 2010, the prevalence of overweight and obesity among adults in Ningbo was around 28 and 7%, respectively (11). In 2016, a study reported a high prevalence of pre-diabetes in rural Ningbo (31%) and identified several risk factors including overweight and obesity (10), indicating the need for early intervention. Currently, no standard weight management programme is available in Ningbo for overweight and obese adults. The main research question to be addressed by the randomized controlled trial (RCT) is whether a weight management programme is effective for overweight and obese adults in Ningbo. The chances of successful completion of a costly RCT improves if the feasibility of its key elements is checked before it starts (12, 13). Thus, the main aim of the current study was to determine the feasibility of undertaking the RCT.

## Methods

### Study Design

A pre- and post-intervention study was conducted to determine the feasibility of undertaking the RCT. The study was guided by the UK's National Institute for Health Research (NIHR) guideline for feasibility studies (13). In this feasibility study, some of the important parameters needed to design the RCT were estimated, such as the recruitment rate, follow-up rates and standard deviation (SD) of the outcome measure (needed to calculate the sample size of the RCT) (13).

### Study Setting

The study was conducted at Ningbo First Hospital, China. Ningbo First Hospital is a tertiary care hospital. Local patients, as well as those from surrounding areas, visit this hospital. The population of the city is around 8.2 million (14). In 2010, the prevalence of overweight among 18–44, 45–59, and ≥60 years old people in Ningbo was around 23, 35, and 27%, respectively. The corresponding figures for obesity were 7, 8, and 7% (11).

### Study Period

The study duration was from 01 July 2015 to 30 September 2017.

### Study Participants and Eligibility Criteria

Those aged 18–65 years of age, with a BMI of ≥24 kg/m^2^ (i.e., overweight/obese) and were willing to comply with the programme were included in the study. Pregnant women, those with cognitive impairment or any serious/uncontrolled medical condition (e.g., cancer) or receiving any related non-pharmaceutical/pharmaceutical intervention were excluded.

### Recruitment and Follow-Ups

The recruitment period was from 01 July 2015 to 31 March 2017. A range of strategies was used to recruit participants, such as advertisements in the local newspapers and social media like WeChat, posters displayed at the outpatient clinic and referrals from the outpatient clinic. People providing written informed consent were screened for eligibility. Eligible participants were enrolled on to the programme. Participants were followed up at 3-month (01 October 2015 to 30 June 2017) and 6-month (01 January 2016 to 30 September 2017).

### Intervention

A multidisciplinary team, consisting of three clinicians, two dieticians, one exercise specialist, and one psychologist, delivered the programme over a period of 3-months. The main aim of the programme was to manage weight through lifestyle modification—diet and physical activity (15). A variety of behavior change methods was used in the programme that focused on: problem solving, goal setting, how to carry out a particular task or activity, planning to provide social support or make changes to the social environment, self-monitoring of weight and behaviors that can affect weight and feedback on performance (2). Table 1 shows the structure of the weight management programme.

**Table 1:** Weight management programme structure.

**Month**	**Session**	**Face-to-face/synchronous online**	**Individual/group**	**Duration**
1	1	Face-to-face	Individual	2 h
1	2	Face-to-face	Group (around 10 participants/group)	2 h
2-3	3 to 22 (first 2 weeks- every day; next 6 weeks- once a week)	Synchronous online (social networking App on mobile)	Individual (feedback on diet and physical activity)	10 min

### Study Parameters, Data Collection, and Statistical Analyses

- Recruitment: number of people who gave written informed consent and were screened for eligibility, and were found eligible and were enrolled on to the programme;
- Follow-ups: number of participants followed-up at 3- and 6-month;
- SD of the outcome measure (i.e., BMI).

The data were collected on age (in years), sex (male and female), education (no qualifications, class 1–6, class 7–12, and university/college), work type (low, moderate, and high physical work) (16), income (<3,000 and ≥3,000 CNY/month) (17) and marital status (married and single/divorced/widowed). A structured questionnaire, developed and tested by the research team, was used for this purpose. Anthropometric measurements (height and weight) were taken according to the standard technique by the research team. Participants stood on an automated height and weight scale (OMRON, HNH-318) with bare feet close together, arms at the side and wore light clothing.

For categorical data, numbers and percentages were reported. For continuous data, summary measures of location (mean) and spread (standard deviation) were reported, as appropriate to the distribution (symmetrical). One-way repeated measures ANOVA was used to compare changes in BMI at 3- and 6-month from the baseline to get initial estimates of effects.

### Sample Size

A formal sample size calculation is not required for a feasibility study (13). However, to get initial estimates of effects, at least 193 participants were needed in the study. This was calculated in relation to the chosen power (80%) and significance level (5%, two-tailed) and the desired small effect size (0.2) (18).

### Ethics

The study was ethically approved by the Research Ethics Committee, Ningbo First Hospital, China.

## Results

**Figure 1:**
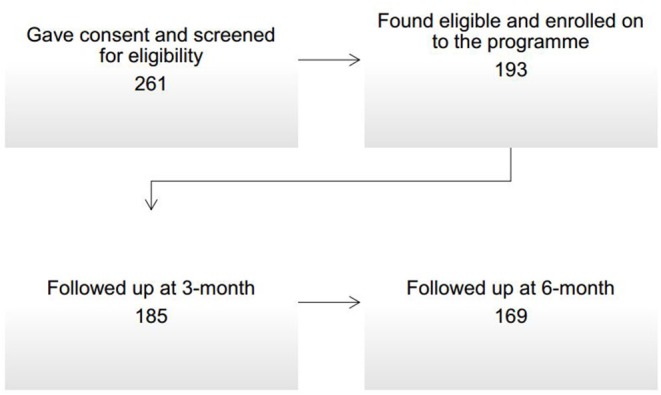
Recruitment and follow-up of participants.

Figure 1 shows the recruitment and follow-up of participants. Two hundred and sixty one people gave written informed consent and were screened for eligibility. Out of these 261 people, 193 (74%) were found eligible and were enrolled on to the programme. Those who enrolled on to the programme, 185 (96%) and 169 (88%) were followed up at 3- and 6-month, respectively.

Table 2 reports the baseline characteristics of participants. The mean age of the participants was 32 years and 64% of them were female. Table 3 reports the change in BMI (mean) at 3- and 6-month from the baseline. The SD of the outcome measure was 4.85. BMI reduced at 3-month follow-up (−1.98, 95% CI −2.19 to −1.79, *p* < 0.001) and at 6-month follow-up (−2.42, −2.71 to −2.11, <0.001).

**Table 2:** Baseline characteristics of participants.

	***N* (%) 193 (100%)**
**Age (years)**	31.5 ± 8.5^*^
**Sex**
Male	70 (36.3%)
Female	123 (63.7%)
**Education**
University/college	145 (75.1%)
Class 7–12	46 (23.8%)
Class 1–6	2 (1.1%)
No qualifications	0
**Work type**
Low physical work	147 (76.2%)
Moderate physical work	11 (5.7%)
High physical work	0
Unknown	35 (18.1%)
**Income (CNY/month)**
<3,000	45 (23.3%)
≥3,000	108 (56.0%)
Unknown	40 (20.7%)
**Marital status**
Married	107 (55.4%)
Single/divorced/widowed	83 (43.0%)
Unknown	3 (1.6%)

* *Mean ± SD*.

**Table 3:** Changes in BMI (mean) at 3- and 6-month from the baseline.

	**Baseline**	**3-month**	**Difference between 3-month and baseline (95% CI)**	***P*-value**	**6-month**	**Difference between 6-month and baseline (95% CI)**	***P*-value**
**BMI (kg/m**^**2**^**)**	32.47 (4.85)^*^	30.44 (4.52)^*^	−1.98 (−2.19 to −1.79)	<0.001	29.76 (4.43)^*^	−2.42 (−2.71 to −2.11)	<0.001

* *SD*.

## Discussion

The feasibility of undertaking the RCT of the weight management programme for overweight and obese adults in Ningbo, China was found to be promising in this study. BMI reduced at 3- and 6-month follow-ups. In the RCT, the primary outcome will be the change in BMI from the baseline to 6-month follow-up. The same intervention will be provided over the same period, as it was found to be feasible to deliver such an intervention and there was some evidence of its efficacy. Similarly, based on the high recruitment and follow-up rates in this study, the same recruitment and follow-up strategies will be used in the RCT. We will try some other recruitment strategies as well, such as referrals from other healthcare providers in Ningbo. In the RCT, the control group participants will receive enhanced standard care i.e., a group session on weight management, delivered by a different team member to avoid contamination. We also intend to do long-term follow-ups in the RCT i.e., annual follow-ups for 2 years. Our sample size estimation suggests that we will need at least 844 participants in the RCT i.e., 422 per group. It will be a two-arm parallel superiority trial. This calculation is based on the type-1 error of 5% (two-tailed), 80% power, 1 kg/m^2^ minimal detectable difference in the mean BMI between the two arms, the standard deviation of 4.85 and loss to follow-up of 12% at 6-month.

Similar feasibility studies on weight management interventions have been conducted among different populations and settings in China and elsewhere (such as in Turkey and Australia) (19–21). Our study was conducted at a tertiary care hospital in China, and the recruitment rate was 74%. The recruitment rate was 89% in the Chinese study conducted among an occupational population, 73% in the Turkish study conducted in primary care among women and 42% in the community-based study conducted in Australia (19–21). In our study, the follow-up rates were 96% at 3-month and 88% at 6-month. In the other Chinese study, it was 86% at 3-month (19). In the Turkish study, the follow-up rates were 50% at 6-month and 40% at 18-month (20). In the Australia study, the follow-up rate was 88% at 3-month (21). The effectiveness of the intervention was proved in the subsequent RCT conducted in Australia (22).

This study has a number of strengths and weaknesses. As far as we are aware, this was the first weight management programme study (feasibility study) in Ningbo. A qualitative study (semi-structured interviews) was also conducted with a sample of participants to explore their experiences in taking part in this programme and study, which will be published separately. Although in this feasibility study, the RCT design would have been the gold standard, the pre- and post-intervention design has provided the estimates of many important parameters needed to design the RCT, if not all. One of the parameters which we couldn't estimate was the willingness of participants to be randomized. The absence of a control group and relatively short-term follow-ups were some of the limitations which we intend to address in the RCT. We used a mobile social networking App to deliver a part of the intervention. Although mobile phones are extensively used in China and mobile phone-based weight management interventions have their own advantages (15), many people including older people may find it difficult to use.

## Data Availability Statement

The dataset will be available upon request unless there are legal or ethical reasons for not doing so.

## Ethics Statement

The study was ethically approved by the Research Ethics Committee, Ningbo First Hospital, China.

## Author Contributions

MX and KC designed the study, analyzed the data, and wrote the first draft of the manuscript. MX, KC, JL, NR, YC, FH, JC, and LL revised it critically for important intellectual content and approved the final version.

### Conflict of Interest

The authors declare that the research was conducted in the absence of any commercial or financial relationships that could be construed as a potential conflict of interest.
